# Oral Microbiota Composition and Function Changes During Chronic Erythematous Candidiasis

**DOI:** 10.3389/fcimb.2021.691092

**Published:** 2021-08-16

**Authors:** Xin Lyu, Hui Zheng, Xu Wang, Heyu Zhang, Lu Gao, Zhe Xun, Qian Zhang, Xuesong He, Hong Hua, Zhimin Yan, Feng Chen

**Affiliations:** ^1^Department of Oral Medicine, Peking University School and Hospital of Stomatology, National Center of Stomatology, National Clinical Research Center for Oral Diseases, National Engineering Laboratory for Digital and Material Technology of Stomatology, Beijing Key Laboratory of Digital Stomatology, Research Center of Engineering and Technology for Computerized Dentistry Ministry of Health, NMPA Key Laboratory for Dental Materials, Beijing, China; ^2^Central Laboratory, Peking University School and Hospital of Stomatology, National Center of Stomatology, National Clinical Research Center for Oral Diseases, National Engineering Laboratory for Digital and Material Technology of Stomatology, Beijing Key Laboratory of Digital Stomatology, Research Center of Engineering and Technology for Computerized Dentistry Ministry of Health, NMPA Key Laboratory for Dental Materials, Beijing, China; ^3^Department of Microbiology, The Forsyth Institute, Cambridge, MA, United States

**Keywords:** oral candidiasis, *Candida albicans*, oral microbiota, metagenomics, functional potentials

## Abstract

Oral microbiota is constantly changing with the host state, whereas the oral microbiome of chronic erythematous candidiasis remains poorly understood. The aim of this study was to compare oral microbial signatures and functional profiling between chronic erythematous candidiasis and healthy subjects. Using shotgun metagenomic sequencing, we analyzed the microbiome in 12 chronic erythematous candidiasis, 12 healthy subjects, and 2 chronic erythematous candidiasis cured by antifungal therapy. We found that the salivary microbiota of chronic erythematous candidiasis was significantly different from that of healthy subjects. Among them, *Rothia mucilaginosa* and *Streptococcus mitis* were the most abundant disease-enriched species (Mann-Whitney U-test, *P* < 0.05). In addition, co-occurrence network analysis showed that *C. albicans* formed densely connected modules with oral bacterial species and was mainly positive connected to *Streptococcus* species. Furthermore, we investigated the functional potentials of the microbiome and identified a set of microbial marker genes associated with chronic erythematous candidiasis. Some of these genes enriching in chronic erythematous candidiasis are involved in eukaryotic ribosome, putative glutamine transport system, and cytochrome bc1 complex respiratory unit. Altogether, this study revealed the changes of oral microbial composition, the co-occurrence between *C. albicans* and oral bacteria, as well as the changes of microbial marker genes during chronic erythematous candidiasis, which provides evidence of oral microbiome as a target for the treatment and prevention of chronic erythematous candidiasis.

## Introduction

Oral microbiota is a reflection of the host state and plays an important role in the development of various diseases. Previous studies have shown us the profiling of human oral microbiota in many oral diseases, such as periodontitis, dental caries, and oral squamous cell carcinoma with the use of 16S rRNA sequence analysis or shotgun whole-genome metagenomic methods (Li et al., 2014; Baker et al., 2021; Sarkar et al., 2021). However, there remains some diseases which are well worth exploring from the perspective of oral microbiota.

Chronic erythematous candidiasis is the most common type of Oral candidiasis (Oral candidosis, OC) (Hu et al., 2020), which is the most common opportunistic fungal disease occurring in oral cavity. It is estimated that 5% of newborns, 10% of elderly patients, 30-94% of individuals with malignant tumors, and nearly 90% of HIV-infected patients can develop into OC (Davies et al., 2008; Poncet et al., 2009). *Candida albicans* (*C. albicans*), a symbiotic microorganism carried by about 80% of the general population, is widely believed to be the main causative agent of OC, and accounts for up to 95% of cases (Vila et al., 2020). A variety of local and systemic factors can lead to the overgrowth of *C. albicans* on oral mucosa, and make it from commensal to pathogenic (Millsop and Fazel, 2016).

Increasing evidence indicates that *C. albicans* exhibits diverse interactions with oral bacterial species, ranging from antagonistic to synergistic (Montelongo-Jauregui and Lopez-Ribot, 2018; Abrantes and Africa, 2020). On the one hand, *C. albicans* was shown to co-aggregate with varieties of oral bacterial flora, such as *Streptococci*, *Fusobacterium nucleatum* and *Porphyromonas gingivalis* (Wu et al., 2015; Montelongo-Jauregui and Lopez-Ribot, 2018; Xiao et al., 2018; Vila et al., 2020). On the other hand, some oral bacteria can affect the colonization and activity of *C. albicans*. For example, *Streptococcus oralis* and *Porphyromonas gingivalis* can enhance the expression level of genes encoding cell surface adhesin in *C. albicans* and the biofilm formation ability of *C. albicans* (Cavalcanti et al., 2016; Bartnicka et al., 2019).

Although various studies have explored the relationship between *C. albicans* and oral bacteria, few studies clarified the whole oral microbiome during *C. albicans* infection, especially in chronic erythematous candidiasis patients without the presence of other factors that can affect the composition of microbiota. In addition, mapping the complex nature of oral microbiota in chronic erythematous candidiasis patients is of great significance in expanding our understanding of oral microbiome and chronic erythematous candidiasis itself. Therefore, this study aimed to compare the salivary microbiota and its gene function between chronic erythematous candidiasis and healthy subjects, in order to profile the microbial communities in chronic erythematous candidiasis.

## Materials and Methods

### Subject Recruitment

Study participants aged 45-65 years were recruited from the Department of Oral Medicine, Peking University School and Hospital of Stomatology, China. Participants were excluded from the research if they (1) had removable dentures, (2) suffered hyposalivation (unstimulated salivary flow rate <1ml/10min) (Lopez-Pintor et al., 2016), diabetes, cancer, anemia, HIV positive, or other severe local or systemic infections, (3) used steroid drugs, immunosuppressants, antibiotics or mouthwash in the last 3 months, (4) had head or neck radiotherapy within the 3 previous months, (5) had smoking history. Participants with clinical manifestations of chronic erythematous candidiasis (Coronado-Castellote and Jimenez-Soriano, 2013) and a positive result on a mycological examination (smear and culture) were included in the disease group (DIS, n = 12). Participants with no clinical manifestations of oral candidiasis, no other oral mucosal disease and a negative result on a mycological examination (smear and culture) were included in the healthy control group (HC, n = 12). Besides, 2 participants suffered from chronic erythematous candidiasis before, but now had been cured by antifungal therapy were also included into our research. The social demographics including age and gender were collected. A comprehensive oral examination including salivary pH, probing depth (PD), DMFT (decayed, missing and filled teeth) was performed by an experienced dentist.

The study protocol was reviewed and approved by the Ethics Committee of the Peking University Health Science Center (PKUSSIRB-2013034). All participants received both written and oral information before consenting to participate in our study.

### Saliva Sample Collection

For DIS and HC group, their salivary samples were collected after taking the questionnaire and completing the initial screening. For the cured group, their salivary samples were collected when the patients were cured and discontinued the antifungal drugs for 1 week. Unstimulated mixed saliva was collected by spitting into a 50-ml sterile collection tube, with at least 2 ml of volume. All participants were asked to skip breakfast and not to do tooth brushing 3 h before saliva collection. Saliva was collected between 8 a.m. and 10 a.m. by a single dentist in a quiet room. Samples were centrifuged at 10,000×g for 20 min at 4°C. Sediments were stored at -80°C until DNA extraction.

### DNA Extraction, Library Preparation, and Whole Genome Shotgun Sequencing

Genomic DNA extraction was performed using the FastDNA SPIN kit for Soil (MP Biomedicals, USA). The DNA concentration was determined with the Nanodrop 8000 (Thermo Scientific, USA), and DNA quality was estimated by agarose gel electrophoresis. Genomic DNA was sheared by the Biorupter ^®^ Pico sonication device (Diagenode, Belgium). DNA fragments of approximately 200 bp were selected with agarose gel electrophoresis. DNA libraries were constructed using the NEBNext Ultra DNA Library Prep Kit for Illumina (Illumina Inc, USA) according to the instruction manual. The insert size of the DNA libraries constructed from salivary samples varied from 155 to 266 bp (mean 210 ± 35.5 bp). All library sequencing was performed with 2×125-bp paired-end on the Illumina HiSeq 2000 platform (Illumina Inc, USA).

### Bioinformatics Analysis of Sequence Data

Sequences with more than 3 ambiguous bases were removed. We screened reads for the minimum percentage of high-quality bases (Q30, >=50%) and trimmed low-quality bases (<Q30) on the terminal end. Paired reads with at least 1 read mapped to the human reference genome (GRCh37/hg19) were removed by SOAP2 software (-m 100 -x 1000).

The filtered clean reads were mapped to a database of predefined single-copy phylogenetic marker genes, with default options embedded in the MOCAT pipeline (Sunagawa et al., 2013). To estimate the *C. albicans* load, paired-end reads were mapped to the *C. albicans* reference genome using BWA with default settings. We simply defined the relative abundance of *C. albicans* as the relative abundance of sequences mapped to the reference genome.

Functional profiling of the microbial community was performed based on the Kyoto Encyclopedia of Genes and Genomes (KEGG) database of gene families and modules. The procedures were as follows: firstly, assembled the quality- and human- filtered WGS sequences into contigs using Soap2; then, detected the Open Reading Frames (ORFs) using Markergene software; finally, mapped the ORFs against protein-coding sequences from the KEGG Orthology using bowtie2.

### Statistical Analysis

The data were presented as mean ± standard error unless otherwise indicated. Univariate statistical analyses were performed using t-test and Mann-Whitney U-test. A *P* value < 0.05 was considered statistically significant.

## Results

### Participants’ Characteristics and General Sequence Information

**Figure 1:**
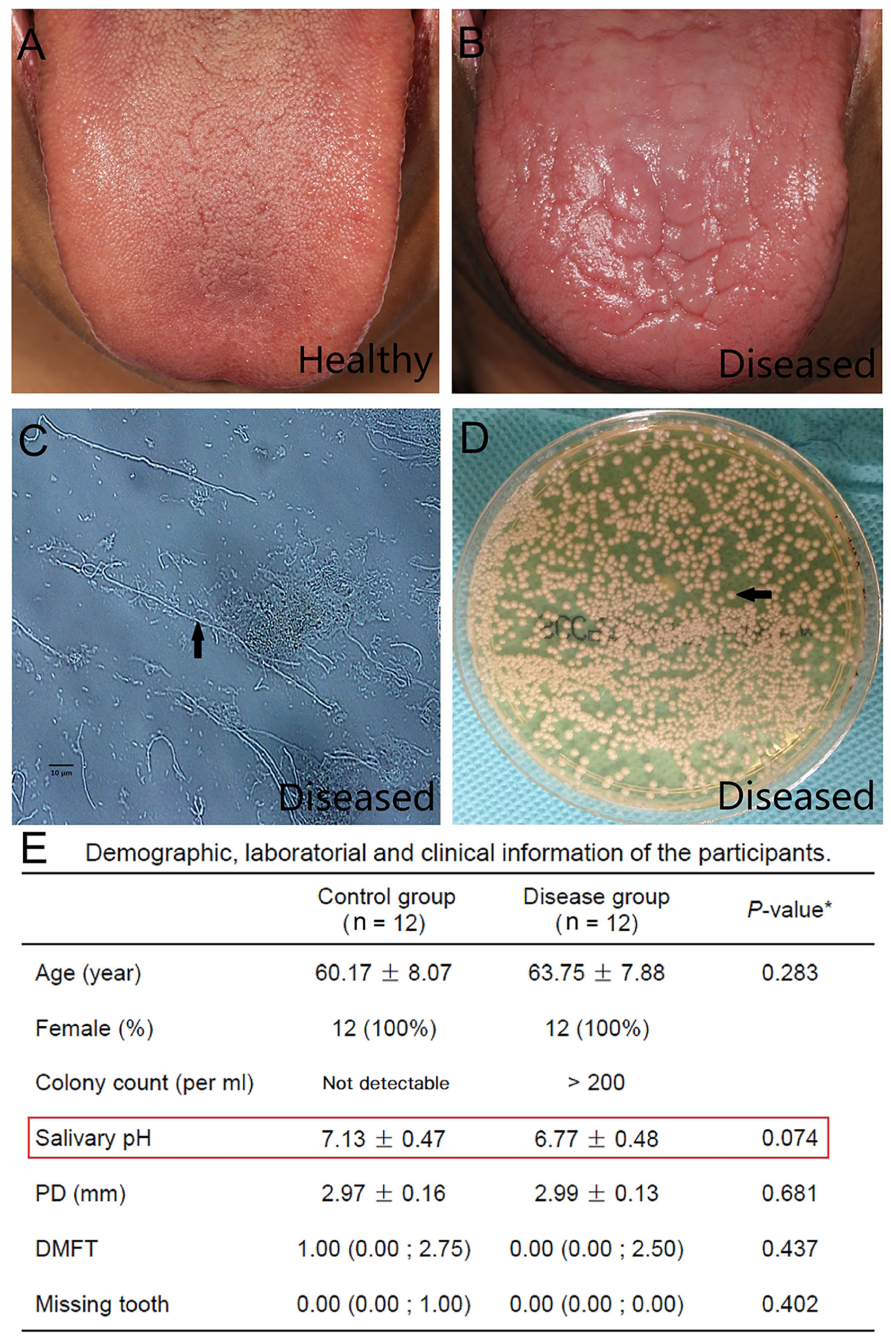
Clinical, laboratory and demographic characteristics of the study participants. **(A)** A clinical picture of the normal dorsum of tongue from a healthy individual. **(B)** A clinical picture of the dorsum of a tongue with chronic erythematous candidiasis from the disease group. Atrophy of the filiform papilla and erythema of the tongue could be observed. **(C)** Many hyphae (arrow) were detected by a smear test using optical microscopy (original magnification ×400). **(D)** A positive result of a salivary culture obtained from Sabouraud’s agar. *Candida albicans* colonies (arrow) could be detected. **(E)** Demographic, laboratory and clinical information of the participants. *Independent t-test for age, salivary pH and PD; Mann-Whitney test for DMFT and missing tooth. The values are the means ± standard deviations, the medians (Q25; Q75) and the numbers of participants (percentage). PD, Probing depth; DMFT, Decayed, missing and filled tooth.

We carried out shotgun metagenomic sequencing of 26 salivary samples, including 12 from healthy subjects (HC group), 12 from chronic erythematous candidiasis patients (DIS group), and 2 from chronic erythematous candidiasis patients cured by antifungal therapy. No significant differences were found in age, salivary pH, PD, DMFT between HC and DIS (**Figure 1** and **Table S1**). It was worth mentioning that salivary pH value showed a decreasing trend in DIS compared with HC, although the difference was not statistically significant (independent t*-*test, *P* = 0.074).

In total, 154.4 gigabases (Gb) of paired-end sequence data were generated with an average of 27.6 million reads (5.5 Gb) per sample. A total of 97.2 ± 0.9% of these reads remained after filtering low-quality reads. Human DNA, which accounted for 48 ± 23.2% (range from 2.7% to 79.5%) of the high-quality reads, was filtered for further processing (**Figure S1**).

### Phylogenetic Analysis of Microbial Community Composition

**Figure 2:**
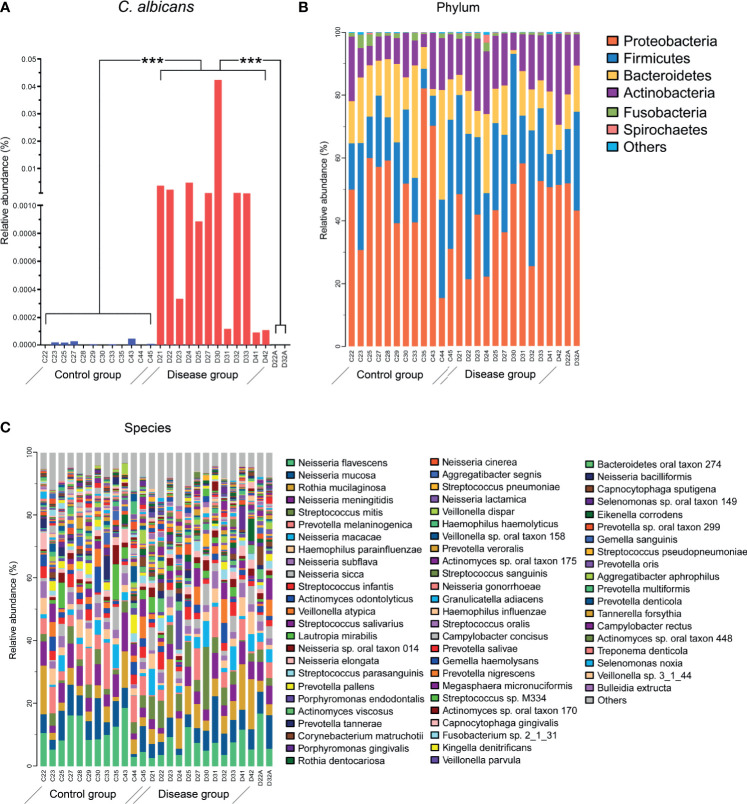
Microbial compositions of salivary samples. **(A)** The relative abundance of *C. albicans* was significantly different between the HC and DIS groups. ***P <0.001. **(B)** The relative abundance levels of bacteria at the phylum levels. **(C)** The relative abundance levels of bacteria at the species levels. Sequence annotation and relative abundance estimation were performed with MOCAT.

The alpha diversity of total microbiota in both DIS and HC was calculated, showing no significant inter-group differentiation (data not shown). In the DIS group, 4.9×10^-3^ ± 12.1×10^-3^% (range from 9.53×10^-5^% to 4.3×10^-2^%) of the metagenomic reads were mapped to the *C. albicans* genome from the NCBI. In HC and in treated samples, sequences homologous to *C. albicans* were barely detected. The relative abundance of *C. albicans* was significantly different between DIS and HC (or cured participants) (**Figure 2A**; independent t-test, *P* < 0.001).

For taxonomic information from the short metagenomic sequences, metagenomic operational taxonomic units (mOTUs) were established based on single-copy phylogenetic marker genes. A total of 443 bacterial species in all samples were identified, which belonged to 9 different phyla (**Figures 2B, C**). The top 20 most abundant species accounted for 65.8% of the total taxa. The salivary community was dominated by *Neisseria* spp., which accounted for 35.9% of the total microbiota. Thirteen species of *Neisseria* were detected, including *N. flavescens* (8.6 ± 4.6%), *N. mucosa* (6.6 ± 3.1%), and *N. meningitides* (5.2 ± 2.4%). Of the 20 most abundant species, 8 belonged to *Neisseria* spp., 4 belonged to *Streptococcus*, and the remaining species belonged to *Prevotella, Rothia, Haemophilus, Actinomyces, Veillonella, Lautropia* and *Porphyromonas*.

### Differences in Salivary Microbiome Between Chronic Erythematous Candidiasis and Controls

**Figure 3:**
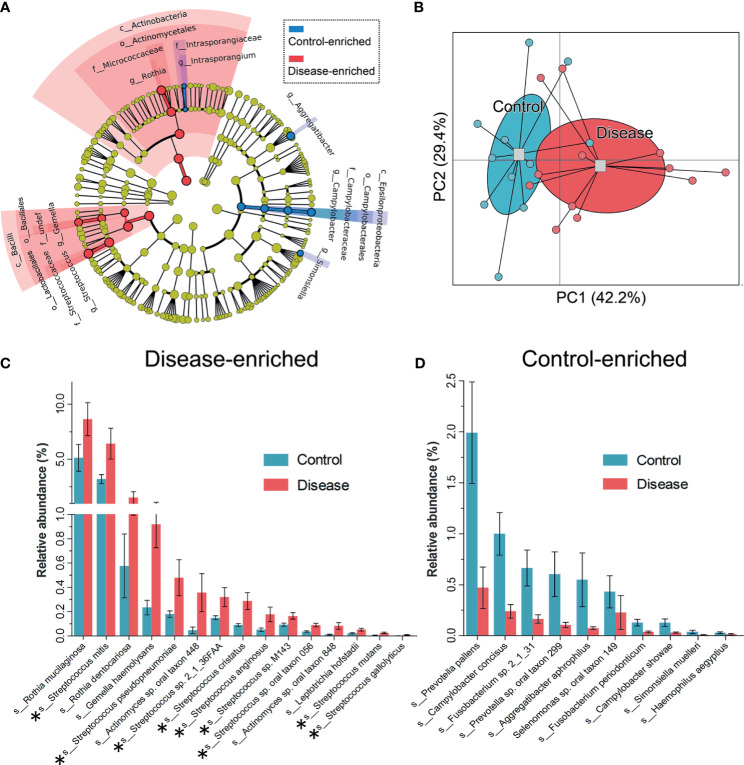
Taxa differentiations between the HC and DIS group. **(A)** Taxa with significant differences in relative abundance at the phylum, class, order, family and genus levels are shown via taxonomic relationships. **(B)** Principal coordinates analysis based on the differentially present taxa using the Bray-Curtis distance. **(C)** Species enriched in the DIS group are shown, the bar represents the mean±sem. Members of *Streptococcus* are marked with “*”. **(D)** Species enriched in the HC group are shown, the bar represents the mean±sem.

Taxa at different taxonomic levels were presented as DIS *versus* HC to describe oral microbial community changes (**Figure 3A**). The microbial communities of HC and DIS were roughly separated in a principal coordinate’s analysis based on differentially present taxa using the Bray-Curtis distance (**Figure 3B**), indicating the distinction between DIS and HC. At the class level, Actinobacteria and Bacilli were more enriched in DIS. At the genus level, *Rothia*, *Gemella* and *Streptococcus* were more enriched in DIS, while *Aggregatibacter*, *Campylobacter* and *Simonsiella* were more enriched in HC (**Figure 3A**). At the species level, 15 species were disease-enriched (*Rothia mucilaginosa* and *Streptococcus mitis* were the most abundant species), and 10 species were control-enriched (*Prevotella pallens* and *Campylobacter concisus* were the most abundant species) (Mann-Whitney U-test, *P* < 0.05) (**Figures 3C, D**). And it’s worth noting that 9/15 disease-enriched species belonged to *Streptococci*.

### *C. albicans* Showed Highly Correlated With *Streptococcus*

**Figure 4:**
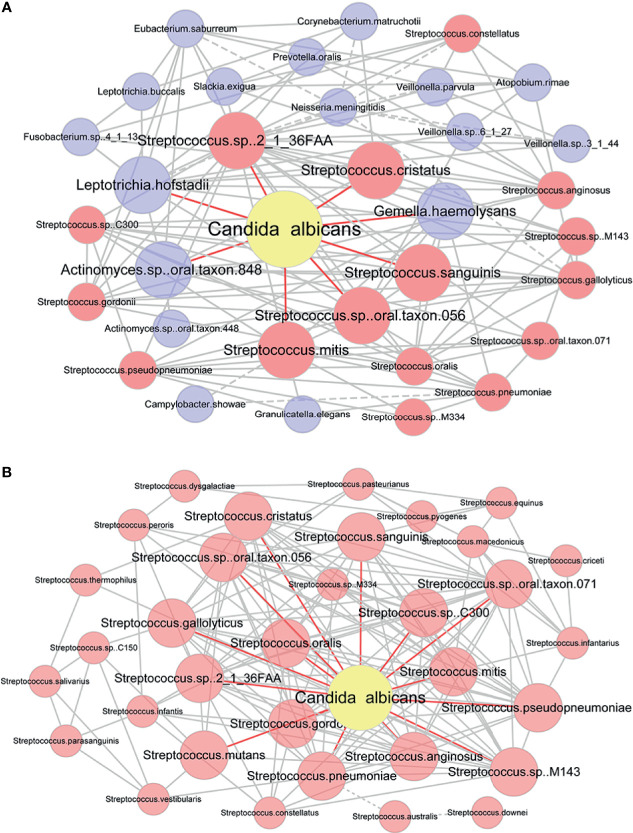
Co-occurrence network of *C. albicans* and bacteria. **(A)** Co-occurrence network modules formed around *C. albicans*. Taxa that were directly correlated with *C. albicans* and those directly corrected with them were used to construct the network. Each pair of nodes connected by an edge was significantly and highly correlated (Spearman correlation test, *P* < 0.05, |r|≥0.6). Solid and dashed lines indicate positive and negative correlations. Taxa that were directly correlated with *C. albicans* are in bold. Nodes that represent members of *Streptococcus* are highlighted in red. **(B)** Co-occurrence network of *C. albicans* and *Streptococcus* spp. Each pair of nodes connected by an edge was significantly and highly correlated (Spearman correlation test, *P* < 0.05). Solid and dashed lines indicate positive and negative correlations. Taxa that were directly correlated with *C. albicans* are in bold.

To analyze the relationship between *C. albicans* and oral bacteria, we constructed a salivary co-occurrence network based on taxonomic datasets of DIS, HC and cured DIS (**Figure S2**). The network consisted of 411 species (92% of the total) and 2625 correlations (edges); 97.2% of the species were positively correlated. We found that *C. albicans* formed densely connected modules with oral bacteria. Among them, 5 of the 8 species that directly correlated with *C. albicans* as well as 11 of the 25 species that indirectly correlated with *C. albicans* were members of *Streptococcus* respectively (Spearman correlation, *P* < 0.05; **Figure 4A**).

Then, correlations between *C. albicans* and *Streptococcus* spp. were evaluated by a newly constructed co-occurrence network (Spearman correlation, *P* < 0.05; **Figure 4B**). Of the 34 *Streptococcus* species detected in saliva to our extent of sequencing, 33 were included in the network and were connected with *C. albicans*. *Streptococcus* spp. and *C. albicans* formed a network with densely connected nodes and formed 338 edges. All correlations between *Streptococcus* spp. and *C. albicans* were positive, indicating a potential mutually promotional relationship.

### Differences in Functional Potentials Between Chronic Erythematous Candidiasis and Controls

To identify the functional role of salivary microbiota and its changes during *C. albicans* infection, we analyzed functional genes using the KEGG database. A total of 6495 KEGG orthologues (KOs) were identified, and the relative abundance levels of KOs were estimated (**Table S2**). The most abundant KOs identified from microbial communities in all samples included genes encoding Bacterial secretion system, ABC transport system, RNA polymerase, Genetic Information Processing, and Aminoacyl-tRNA biosynthesis.

**Figure 5:**
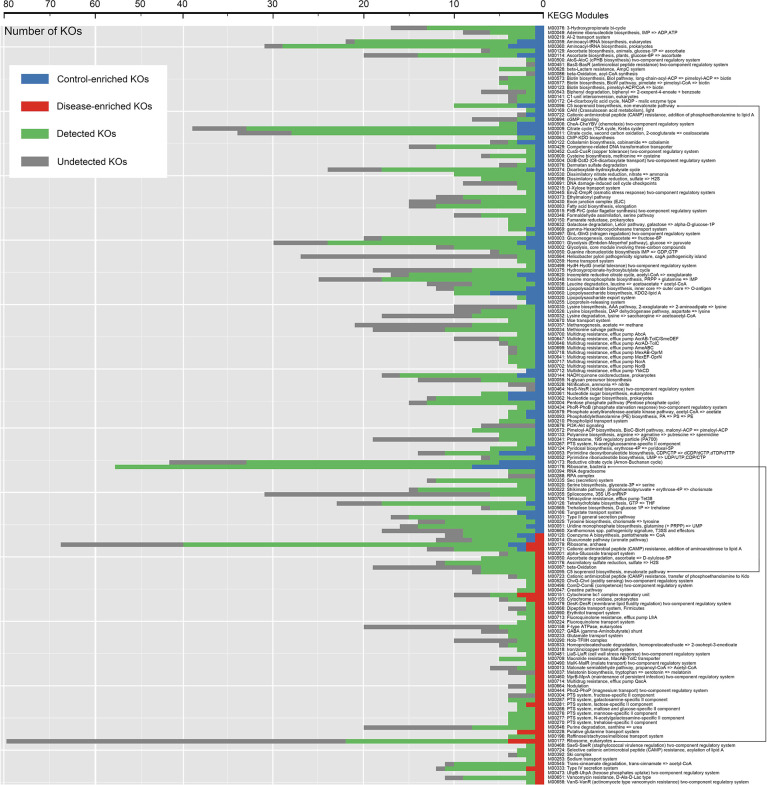
Distribution of KEGG modules for HC-enriched and DIS-enriched KEGG orthologues (KOs). Each bar represents the number of KOs involved in each module. Green bars represent KOs detected in salivary samples to the extent of our sequencing. Blue and red bars represent KOs that were differentially detected in samples from healthy controls and patients with chronic erythematous candidiasis.

Under the criteria (Mann-Whitney U-test, *P* < 0.05), 688 KOs (≈10% of the total we detected) showed significantly different abundance levels in DIS compared to those in HC metagenomes, with 202 enriched KOs and 486 depleted KOs in DIS (**Table S2**). As shown in **Figure 5**, KOs representing lipopolysaccharide biosynthesis system, reductive citrate cycle, bacterial ribosome were enriched in HC, while KOs representing eukaryotic ribosome, putative glutamine transport system, cytochrome bc1 complex respiratory unit were enriched in DIS. The results revealed that the genetic phenotypes of metabolism and other important physiological functions had changed significantly in oral microbiome of chronic erythematous candidiasis.

## Discussion

The Human Microbiome Project (HMP) has been carried out over ten years, and it was found that the complex and common host–microbiome interactions play an important role in the health and various diseases of human (Proctor et al., 2019). Oral microbiome is one of the most diverse microbiomes in human body. Though its composition has been explored in many oral diseases, there are still unexplored areas worth investigating (Wade, 2013). In the present study, we profiled the salivary microbiome of chronic erythematous candidiasis patients with the use of shotgun metagenomic sequencing technique and compared it with that of healthy participants. It was demonstrated that the composition and gene function of salivary microbiota had changed a lot during chronic erythematous candidiasis. What’s more, a strong correlation between *C. albicans* and *Streptococcus* could be found. This project is a complement of the previous studies in oral microbiomes under the disease state. More importantly, it may provide some potential targets for the therapy and prognosis of chronic erythematous candidiasis.

Great attention has been paid to the relationship between *Candida* and oral microflora in different host states. Using 454 pyrosequencing, Kraneveld EA et al. revealed that oral *Candida* load was correlated negatively with the diversity of the salivary microbiome and positively with the relative abundance of *Streptococci* in older Dutch adults (Kraneveld et al., 2012). With 16s rRNA amplicon sequencing, Xiao J et al. found that *C. albicans* influenced the composition and diversity of oral bacterial community in the context of severe early childhood caries (Xiao et al., 2018). By the use of 16s rRNA gene sequencing, Bertolini M et al. demonstrated that *C. albicans* infection was associated with loss of mucosal bacterial diversity in both oral and small intestinal mucosa in a mouse intravenous chemotherapy model (Bertolini et al., 2019). However, few studies explored the oral microbiome during chronic erythematous candidiasis without the presence of other factors that can affect the composition of microbiota.

Thus, in the present study, shotgun metagenomic sequencing technique was used to depict the salivary microbiome of chronic erythematous candidiasis patients. What’s more, by adopting stringent inclusion criteria, many interference factors that can influence oral microbiota, such as wearing removable dentures and systemic diseases (O’Donnell et al., 2015), were eliminated. As a result, we collected a more valuable and reliable data on the interaction between *C. albicans* and oral microflora.

The composition of salivary microbiota showed significant difference between chronic erythematous candidiasis and healthy subjects. We found that *Prevotella rallens* was the most abundant species in healthy participants and significantly decreased in chronic erythematous candidiasis. It was reported that *Prevotella* spp. in all oral sites of clinically healthy individuals are part of the core microbiome (Ishaq et al., 2017). Therefore, changes in the composition of *Prevotella* genus may serve as a signal of a potential observational index for assessing or providing a prognosis for *C. albicans* infection and other unhealthy states. On the contrary, *Rothia mucilaginosa* and *Streptococcus mitis* were the most abundant disease-enriched species. Both bacteria are normal inhabitant of the human oral cavity and their abundance changed a lot in some abnormal status of oral cavity. It has been reported that the abundance of *Rothia mucilaginosa* was significantly increased in tongue leukoplakia lesions (Amer et al., 2017) and smokeless tobacco users (Halboub et al., 2020). And the abundance of oral *Streptococcus mitis* was reported to have changed a lot in periodontitis patients (Lundmark et al., 2019). In addition, the relative abundance of *Streptococcus*, *Rothia* and *Gemella* was increased in chronic erythematous candidiasis, but decreased after treatment (the 22nd and 32nd sample), which indicates that *Candida* infection has affected the native oral microbiome. Among them, the relative abundance of *Gemella* is also changed in an unhealthy oral cavity and is reported to be higher in erosive oral lichen planus (Yu et al., 2020). Hence, these microorganisms changing during chronic erythematous candidiasis may play potential significant roles in the disease process.

Therefore, we further analyzed the co-occurrence network between *C. albicans* and oral bacteria, and demonstrated the positive correlations between *Streptococcus* spp. and *C. albicans*. Although previous studies investigated the interactions between oral *Streptococcus* spp. and *C. albicans*, they had been focusing on certain species of *Streptococcus* (Ellepola et al., 2019; Abrantes and Africa, 2020). As what has been summarized in a review (Montelongo-Jauregui and Lopez-Ribot, 2018), 7 *Streptococcus* spp. associated with *C. albicans* were listed, all of them could be found in our study showing the correlations between *C. albicans* and 33 *Streptococcus* species. In addition, the results of this study can well represent the *in vivo* and the clinical environment, which is a supplement and extension to the previous studies in the influence of *C. albicans* infection on bacterial dysbiosis in mice model (Bertolini et al., 2019).

Some studies have also explored the underlying mechanisms of the correlations between *Streptococcus* spp. and *C. albicans*. *C. albicans* can positively influence the growth and biofilm formation of *Streptococcus* spp., through different but connected factors and mechanisms, such as fatty acids, carboxylic acids, farnesol and glucans (Metwalli et al., 2013; Ellepola et al., 2019). In addition, *C. albicans* can also induce the expression of virulence genes (e.g., *gtfB, fabM*) in *Streptococcus mutans* (Falsetta et al., 2014). On the other hand, *Streptococci* are considered to play an important role in establishing *C. albicans* colonization. *Streptococcus gordonii*, *Streptococcus oralis*, *Streptococcus sanguis* and *Streptococcus mutans* had been found to be structurally associated and co-aggregate with *C. albicans*, facilitated its yeast-to-hypha transition, and promoted its morphogenesis progress, adherence, colonization and biofilm formation through different mechanisms, such GlcNAc, lactate, H_2_O_2_, peptidoglycan, and Al-2 (Diaz et al., 2012; Metwalli et al., 2013; Dutton et al., 2016).

In our study, 9 of the 15 species enriched in DIS belonged to *Streptococcus* spp., and the co-occurrence network analysis revealed positive correlations between *Streptococcus* spp. and *C. albicans.* In order to support this result, we carried out an *in vivo* mice experiment. The mice were infected on their tongue with *C. albicans* only or co-infected with *C. albicans* and *Streptococcus mutans* for 5 days. We found that co-infection with *C. albicans* and *Streptococcus mutans* triggered significantly greater weight loss than infection with *C. albicans* only, and *Streptococcus mutans* enhanced the colonization of *C. albicans* on mice tongues (**Figure S3**). The above results indicated the interactions between *Streptococcus* spp. and *C. albicans* may serve as an important role in the occurrence and development of chronic erythematous candidiasis. However, more detailed animal or *in vitro* experiments about the relationships between *C. albicans* and key bacteria changed in chronic erythematous candidiasis should be carried out in the future.

In addition, the gene function of salivary microbiome was different between chronic erythematous candidiasis and healthy subjects. It was obvious that the putative glutamine transport system and cytochrome bc1 complex respiratory unit were enriched in DIS. Glutamine transport system is an important part of the nitrogen assimilation metabolism of microorganism. Nitrogen and nicotinate/nicotinamide metabolic pathways had been confirmed to be involved in *C. albicans* morphogenesis, such as filament formation which plays an important role in penetrating endothelial tissue (Han et al., 2019). Cytochrome bc1 (Complex III) is an important part of the electron respiratory transport chain in organisms. The disruption of electron transport chain function increased intracellular levels of reactive oxygen species in yeast. And, the inhibition of cytochrome bc1 could significantly increase the sensitivity of *C. albicans* to photodynamic therapy (Chabrier-Rosello et al., 2010). Basing on the changes in energy metabolism, respiratory transport chain and other biological functions of oral microbiome, we might find some new drug targets for treating chronic erythematous candidiasis.

In conclusion, our study revealed the oral microbial changes occurring in chronic erythematous candidiasis. Close relationships were seen between *C. albicans* and oral bacteria, especially *Streptococcus*. In addition, functional potentials of oral microbiome also changed during chronic erythematous candidiasis, which may provide us with some new insights into the role of oral microecology in pathological and clinical manifestations of chronic erythematous candidiasis.

## Data Availability Statement

The data presented in the study are deposited in the China National GeneBank (CNGB) repository, accession number CNP0001917.

## Ethics Statement

The studies involving human participants were reviewed and approved by Ethics Committee of the Peking University Health Science Center. The patients/participants provided their written informed consent to participate in this study. The animal study was reviewed and approved by Ethics Committee of the Peking University Health Science Center. Written informed consent was obtained from the individual(s) for the publication of any potentially identifiable images or data included in this article.

## Author Contributions

XL: Writing original draft, sample collection, experimentation, data analysis, and data visualization. HuZ: Data analysis, Data visualization. XW: Data analysis, Data visualization. HeZ: Data analysis, Data visualization. LG: Data visualization. ZX: Experimentation. QZ: Experimentation. XH: Data analysis. HH: Sample collection. ZY: Conceptualization, supervision, data interpretation, data analysis, and sample collection. FC: Supervision, data interpretation, and experimentation. All authors contributed to the article and approved the submitted version.

## Funding

This work was financially supported by the National Natural Science Foundation of China (Nos. 81000441, 81570985 and 81991501).

## Conflict of Interest

The authors declare that the research was conducted in the absence of any commercial or financial relationships that could be construed as a potential conflict of interest.

## Publisher’s Note

All claims expressed in this article are solely those of the authors and do not necessarily represent those of their affiliated organizations, or those of the publisher, the editors and the reviewers. Any product that may be evaluated in this article, or claim that may be made by its manufacturer, is not guaranteed or endorsed by the publisher.
