# Supplementary material for: Oral Microbiota Composition and Function Changes During Chronic Erythematous Candidiasis
Source: Front Cell Infect Microbiol. 2021 Aug 16;11:691092. doi: 10.3389/fcimb.2021.691092 (PMC8418087; doi:10.3389/fcimb.2021.691092)
Supplement: Supplementary file 1 [file DataSheet_1.docx]

**Oral** **Microbiota Composition and Function Changes during Chronic Erythematous Candidiasis**

Xin Lyu^1,2†^, Hui Zheng^3,4†^, Xu Wang^1,3†^, Heyu Zhang^3†^, Lu Gao^3^, Zhe Xun^3^,

Qian Zhang^3^, Xuesong He^5^, Hong Hua^1^, Zhimin Yan^1*^, Feng Chen^3*^

^1^ Department of Oral Medicine, Peking University School and Hospital of Stomatology, National Center of Stomatology, National Clinical Research Center for Oral Diseases, National Engineering Laboratory for Digital and Material Technology of Stomatology, Beijing Key Laboratory of Digital Stomatology, Research Center of Engineering and Technology for Computerized Dentistry Ministry of Health, NMPA Key Laboratory for Dental Materials, Beijing, China

^2^ Department of Oral Medicine, Beijing Stomatological Hospital, Capital Medical University, Beijing, China (Present Address)

^3^ Central Laboratory, Peking University School and Hospital of Stomatology, National Center of Stomatology, National Clinical Research Center for Oral Diseases, National Engineering Laboratory for Digital and Material Technology of Stomatology, Beijing Key Laboratory of Digital Stomatology, Research Center of Engineering and Technology for Computerized Dentistry Ministry of Health, NMPA Key Laboratory for Dental Materials, Beijing, China, Beijing, China

^4^ Department of Orthodontics, School and Hospital of Stomatology, Fujian Medical University, Fuzhou, China (Present Address)

^5^ Department of Microbiology, The Forsyth Institute, Cambridge, Massachusetts, USA

Corresponding authors:

Feng Chen: 22# ZhongguancunNandajie, Haidian District, Beijing 100081, China. Email: chenfeng2011@hsc.pku.edu.cn;

Zhimin Yan: 22# ZhongguancunNandajie, Haidian District, Beijing 100081, China. Email: [yzhimin96@163.com](mailto:yzhimin96@163.com).

^†^These authors have contributed equally to this work and share first authorship


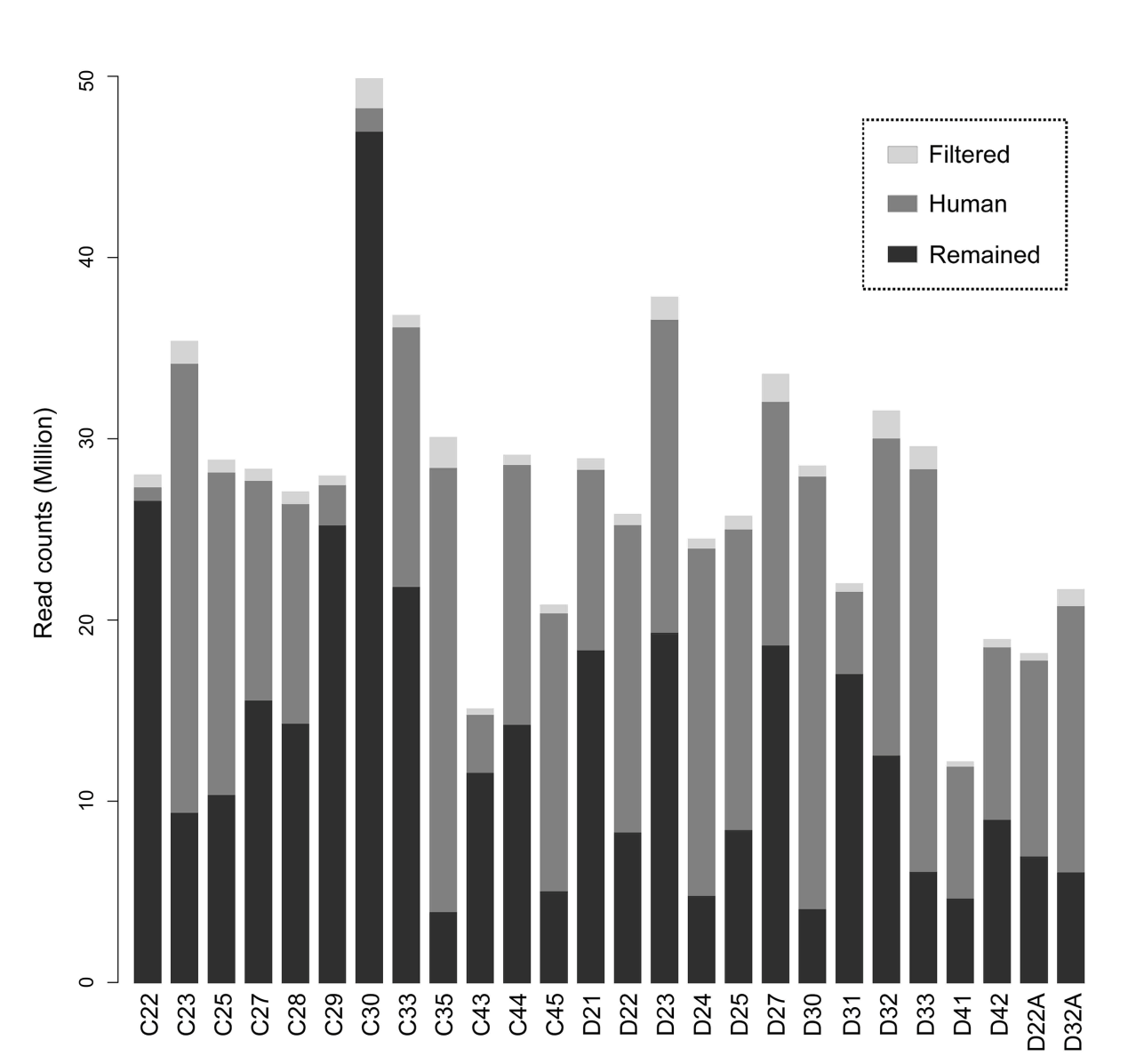


**Figure S1.** Shotgun sequences generated from Illumina Hiseq 2000 platform. Reads of low-quality or classified as human sources were indicated.


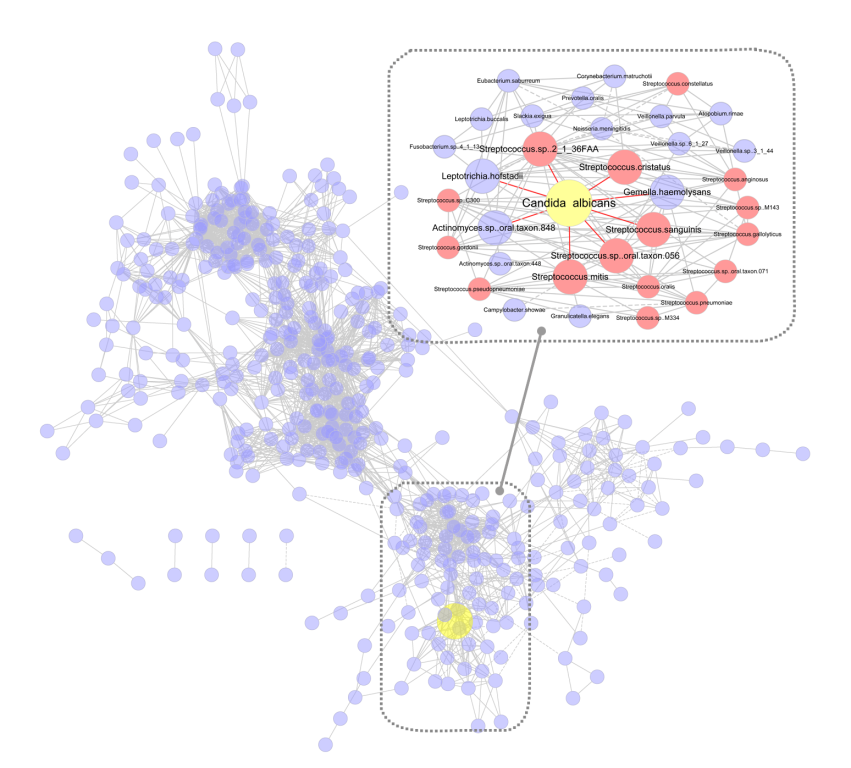


**Figure S2.** Co-occurrence network constructed by all taxa presented in salivary samples. Each pair of nodes connected with edge were significantly and highly correlated (Spearman correlation test, *P* <0.05, |r|≥0.6). Solid and dashed lines indicate positive and negative correlations.


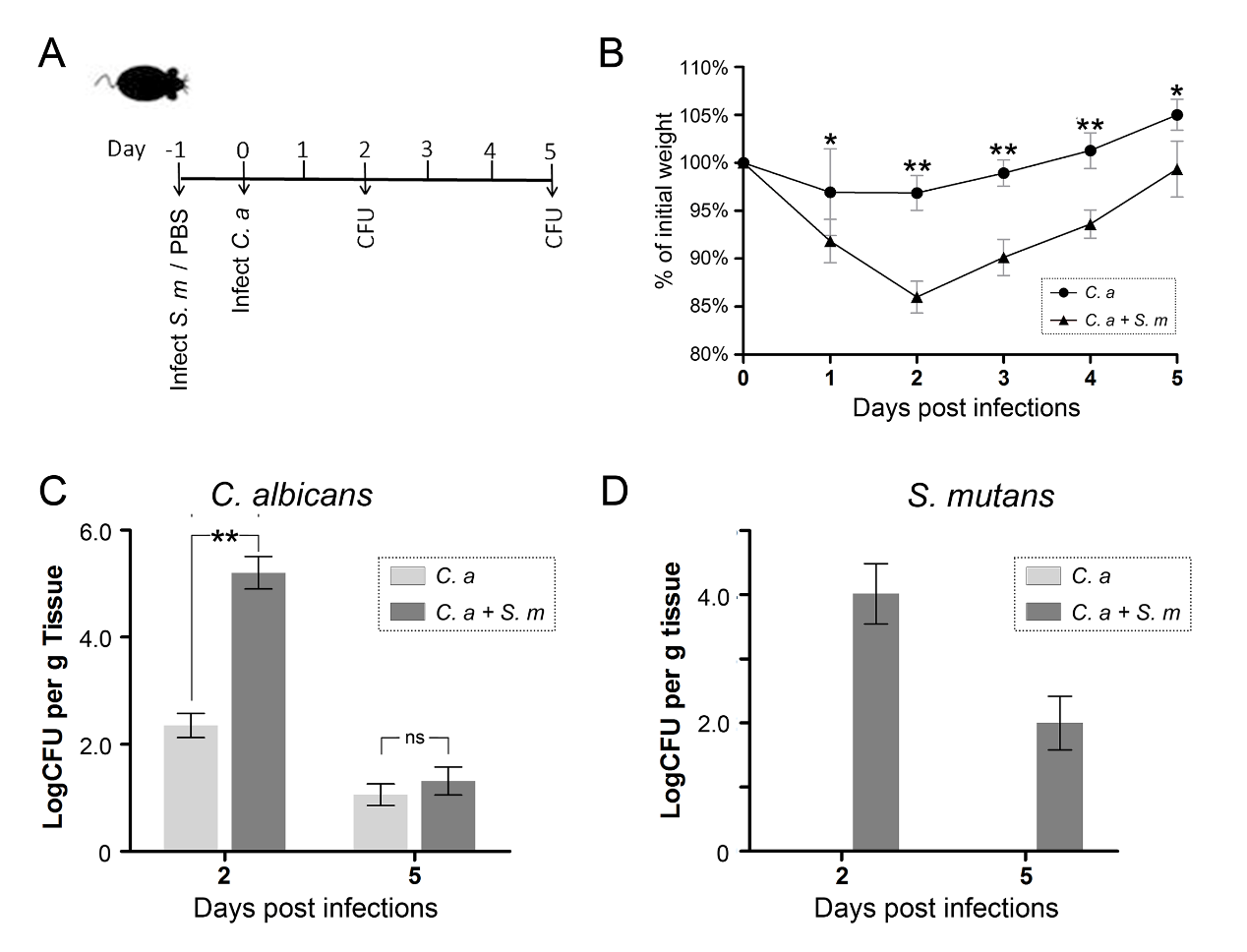
**Figure S3.** Mouse oral co-infection model. *C. albicans* (strain SC5314) was cultured in yeast extract peptone dextrose (YPD) medium at 30˚C for 15-18 h. *S. mutans* (strain UA159) was cultured in brain heart infusion (BHI) medium at 37°C under aerobic, static conditions. C57BL/6 mice were maintained under specific pathogen-free (SPF) conditions and fed with 5% sucrose water. Mice aged 6-8 weeks were randomly placed into 2 groups: *C. albicans*-infected group (n=6) and *S. mutans* plus *C. albicans*-infected group (n=6). On the first day, the mice were anesthetized with chloral hydrate (400 mg/kg) and infected with *S. mutans* (3×10^8^ CFU) or PBS sublingually. On the second day, the mice were anesthetized, and a small cotton swab soaked with 100 μl of *C. albicans* suspension (6×10^8^ cfu/ml) was used for infection without immunosuppression. After 2 and 5 days, the mice were screened for *S. mutans*and *C. albicans* infections. For cfu determination, tongues were excised and homogenized. Undiluted and diluted homogenates were plated on chloramphenicol-supplemented Sabouraud dextrose agar or Mitis Salivarius Agar plus Bacitracin plates for *C. albicans* and *S. mutans* cfu counts.

(A) Timeline of the infection model. (B) Co-infection with *S. mutans* induced more weight loss (n=6, * *P* <0.01, ** *P* <0.01). (C) *S. mutans* increased the *C. albicans* burden on mouse tongues (cfu counts on days 2 and 5, n=6, ** *P* <0.01). (D) *S. mutans* on mouse tongues were counted on day 2 and 5.
